# A Synonymous Variant c.579A>G in the ETFDH Gene Caused Exon Skipping in a Patient With Late-Onset Multiple Acyl-CoA Dehydrogenase Deficiency: A Case Report

**DOI:** 10.3389/fped.2020.00118

**Published:** 2020-03-27

**Authors:** Guorui Hu, Jingxia Zeng, Chunli Wang, Wei Zhou, Zhanjun Jia, Jun Yang, Bixia Zheng

**Affiliations:** ^1^Department of Gastroenterology, Children's Hospital of Nanjing Medical University, Nanjing, China; ^2^Department of Emergency/Critical Care Medicine, Children's Hospital of Nanjing Medical University, Nanjing, China; ^3^Nanjing Key Laboratory of Pediatrics, Children's Hospital of Nanjing Medical University, Nanjing, China

**Keywords:** multiple acyl-CoA dehydrogenase deficiency, whole exome sequencing, ETFDH, synonymous variant, exon skipping

## Abstract

**Background:** Multiple acyl-CoA dehydrogenase deficiency (MADD) is an autosomal recessive disorder characterized by a wide range of clinical features, including muscle weakness, hypoglycemia, metabolic acidosis, and multisystem dysfunctions. Loss-of-function mutations in the electron transfer flavoprotein dehydrogenase (ETFDH) gene are associated with MADD. Disease-causing synonymous variants in the *ETFDH* gene have not been reported so far.

**Methods:** We reported the clinical course of a Chinese girl who was diagnosed with late-onset MADD by the whole exome sequencing. The effects of variants on mRNA splicing were analyzed through transcript analysis *in vivo* and minigene splice assay *in vitro*.

**Results:** The 6-month-old girl initially showed muscle weakness, muscular hypotonia, mild myogenic damage, and fatty liver. The blood and urine metabolic screening by tandem mass spectrometry suggested MADD. Molecular analysis of *ETFDH* gene revealed two novel heterozygous variants, a frameshift mutation c.1812delG (p.V605Yfs^*^34) in exon 13 and a synonymous variant c.579A>G (p.E193E) in exon 5. The transcript analysis *in vivo* exhibited that the synonymous variant c.579A>G caused exon 5 skipping. The minigene splice assay *in vitro* confirmed the alteration of *ETFDH* mRNA splicing which could lead to the production of a truncated protein. Supplementation of riboflavin, carnitine and low-fat diet improved the clinical symptoms.

**Conclusion:** We firstly report a rare case of MADD with a pathogenic synonymous variant in the *ETFDH* gene which highlights the importance and necessity of bioinformatic analysis and functional testing for synonymous variants when searching for causative gene mutations. The results expand the spectrum of pathogenic variants in MADD.

## Introduction

Multiple acyl-CoA dehydrogenase deficiency (MADD; OMIM #231680), also known as glutaric acidemia II or glutaric aciduria II, is an autosomal recessive inherited disorder of fatty acid, amino acid, and choline metabolism. MADD is divided into three clinical types: the neonatal onset form with (type I) or without (type II) congenital anomalies, and the late-onset form (type III) (1, 2). The neonatal-onset forms are usually fatal characterized by severe non-ketotic hypoglycemia, metabolic acidosis, multisystem involvement, and excretion of large amounts of fatty acid- and amino acid-derived metabolites, while the late-onset form is milder and more variable characterized by recurrent episodes of hypoglycemia, metabolic acidosis, vomiting, and muscle weakness during catabolic stress (3). Loss-of-function mutations in the electron transfer flavoprotein dehydrogenase (ETFDH) gene are associated with MADD (4–6). Up to date, according to the Human Genome Mutation Database (HGMD), ~200 mutations have been described in *ETFDH* gene in which synonymous variants have not been reported.

Here we firstly report a case of late-onset MADD carrying a novel synonymous variant c.579A>G (p.E193E) in the *ETFDH* gene and demonstrate that this variant is associated with abnormal mRNA processing leading to exon skipping.

## Methods

### Clinical Presentation

The proband was a 6-month-old girl who was transferred to our pediatric intensive care unit due to muscle weakness for 5 days. She was the second child of non-consanguineous Chinese healthy parents. Her sister was 4.5 years old and had been healthy so far. The proband was born full-term with a weight of 2.7 kg [standard deviation score (SDS) −1.42] and was healthy during the neonatal period. It was her first hospitalization for muscle weakness. She presented progressive arm, lower limb and neck flexors weakness, crying faintly, vomiting, and appetite loss.

Physical examination revealed that the muscle strength was grade 3–4 accompanied with muscular hypotonia. There is no hepatosplenomegaly, facial dysmorphism, and malnutrition with her weight of 6.8 kg (−1.2 SDS). Laboratory tests revealed abnormal liver function (alanine aminotransferase 58 U/L, cutoff < 40; aspartate aminotransferase 152 U/L, cutoff < 40), myocardial damage (troponin I 0.04 ng/ml, cutoff < 0.03; creatine kinase MB 75.5 ng/ml, cutoff < 5; brain natriuretic peptide 552 pg/ml, cutoff < 100) and moderate anemia (hemoglobin 89 g/L). The arterial blood gas analysis showed pH 7.44 with base excess −12.1 mmol/L. Hypoglycemia and electrolyte abnormality were not detected. The lumbar puncture was performed showing no abnormalities in cerebrospinal fluid. The magnetic resonance imaging of head and spine was normal. As her blood creatine kinase (768 U/L, cutoff < 173) and myoglobin (576 ng/ml, cutoff < 56.5) were obvious elevated, the electromyogram was conducted suggesting that there was likely early or mild myogenic damage. In addition, the computed tomography scan indicated fatty liver. Considering the possibility of genetic metabolic diseases, multivitamins, and L-carnitine (100 mg/kg/day) were administered. Meanwhile, the blood and urine metabolic screening by tandem mass spectrometry indicated MADD.

A high dose of riboflavin (150 mg/day) was started immediately after obtaining the abnormal metabolic results, with a low-fat diet. The muscle strength and muscle tone of the patient progressively increased. The patient was discharged after 19 days in hospital with her muscle strength grade 4–5.

### Genetic Analysis

Peripheral blood samples were collected from the patient and her family and sent to Chigene Translational Medical Research Center Co. Ltd. (Beijing, China) for whole exome sequencing. All variants were denoted based on the NCBI reference sequence for *ETFDH* (NM_004453).

### Bioinformatics Predictions

The change of amino acid sequence of protein caused by DNA variant was predicted by the MutationTaster program. To analyze the potential effect of synonymous variant on putative splicing regulatory elements as exonic splicing enhancer (ESE) and exonic splicing silencer (ESS), we used Human Splicing Finder program.

### Transcript Analysis *in vivo*

Total RNA was extracted from peripheral leukocytes of the proband and parents. We used mRNA extracted from a healthy volunteer blood sample to obtain normal control leukocyte. RNA was isolated with the QIAGEN miRNeasy Mini Kit, and then reverse-transcribed into cDNA by using random hexamers and the SuperScript III transcriptase (Invitrogen). Reverse transcription PCR (RT-PCR) was performed in 25 μl volumes using 100 ng of each cDNA as template. PCR products were separated on 1.5% agarose and sequenced with an ABI 3130 genetic analyzer (Applied Biosystems).

### Minigene Splice Assay *in vitro*

Genomic DNA was isolated from peripheral leukocytes under standard methods using a DNA isolation kit (Tiangen, China). We used pSPL3 vector, a generous gift from Dr. Irene Bottillo (University of Sapienza, Italy) and Dr. Leping Shao (University of Qingdao, China). Patient DNA carrying the synonymous variant was used to generate genomic fragments of *ETFDH* including exon 5 with ± 150 bp of its upstream and downstream intronic sequences. Both edges of the shortened introns were properly designed by the Human Splicing Finder program so as to avoid the activation of cryptic splicing. The primers used in this study were edited by Primer5 software (Forward:5′-accagaattctggagctcgagTAGCCTTGTAAGCCAAGCAG-3′; Reverse: 5′-atcaccagatatctgggatccTTGTCCTTGGAAGAGGGCAT-3′). All of the indicated fragments were cloned into a pSPL3 vector with the XhoI and BamHI using ClonExpressTM II One Step Cloning Kit (Vazyme Biotech Co., Ltd). All constructs were sequenced to confirm the distinct mutation and absence of off-target mutations in the constructs.

HEK293T cells were cultivated in Dulbecco's Modified Eagle's Medium (DMEM) at 37°C in 5% CO_2_. Cells were transfected 24 h after seeding in 12-well plates with 1 μg DNA of constructs containing wild or mutant alleles using Lip2000 (Invitrogen). Twenty-four hours after transient transfection, total RNA was extracted from cells using Trizol Reagent (Takara, Japan). First cDNA strand was reversely transcribed using the SuperScript III transcriptase (Invitrogen) together with random hexanucleotide primers. One hundred nanograms of each cDNA were amplified by TaKaRa Ex Taq (Takara, Japan) with primers located in the two cassette exons of pSPL3 vector. The primers sequences as follows: Forward SD 5′-TCTGAGTCACCTGGACAACC-3′ and reverse SA 5′-ATCTCAGTGGTATTTGTGAGC-3′. PCR products were analyzed by agarose gel electrophoresis and proven by sequencing of extracted DNA. Gel was containing 1.5% agarose in Tris-acetate-EDTA buffer.

## Results

### Genetic Findings

**Figure 1:**
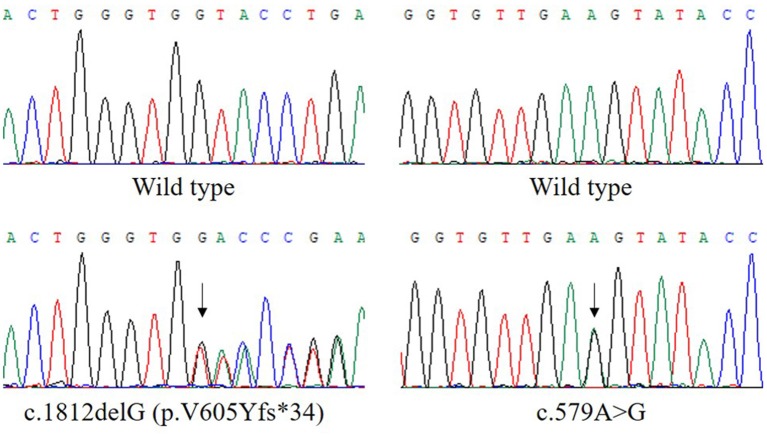
The compound heterozygous variants in the ETFDH gene detected in our patient. The two variants, c.1812delG (p.V605Yfs*34) and c.579A>G, were inherited from her mother and father, respectively. The reference transcript is NM_004453.

Analysis of exome sequencing data identified compound heterozygous variants in the *ETFDH* gene: c.1812delG (p.V605Yfs^*^34) and c.579A>G which were inherited from her mother and father, respectively (Figure 1). The deletion variant c.1812delG (p.V605Yfs^*^34) in exon 13 was predicted to lead to a frameshift after codon 604. The other variant c.579A>G (p.E193E) in exon 5 was predicted to be a synonymous variant which didn't change the amino acid Glu at the position 193 of ETFDH protein sequence. These two variants were found in neither the Genome Aggregation Database (gnomAD) nor HGMD. According to the American College of Medical Genetics and Genomics (ACMG) guidelines (7), the variant c.1812delG (p.V605Yfs^*^34) could be classified as pathogenic.

### Bioinformatics Prediction

**Figure 2:**
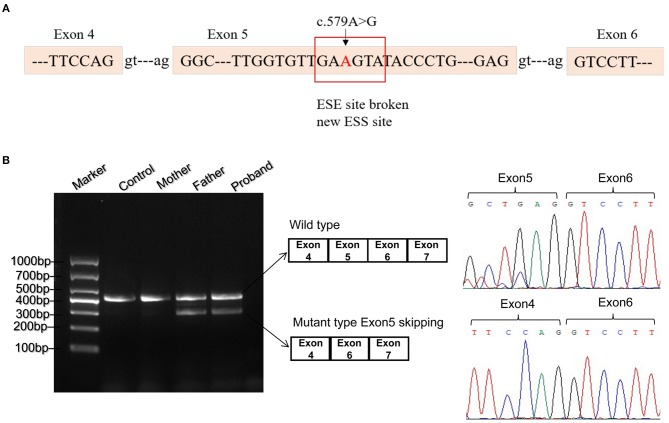
Bioinformatics predictions and transcript analysis *in vivo* for the synonymous variant c.579A>G. **(A)** Human Splicing Finder program predicted that c.579A>G might affect auxiliary cis-acting splicing regulatory elements including destroying the original exonic splicing enhancer (ESE) and generating a new exonic splicing silencer (ESS). **(B)** Gel electrophoresis of RT-PCR fragments *in vivo* showed that c.579A>G caused abnormal mRNA splicing leading to two transcripts differed by about 100 bp. Sequence analysis of the cDNA showed that the shorter transcript lacked a sequence corresponding to exon 5 of the *ETFDH* gene.

As missense or synonymous variants might disrupt splice sites, Human Splicing Finder program was used to analyze the potential effects of the synonymous variant c.579A>G on mRNA splicing. As a result, this variant might affect auxiliary cis-acting splicing regulatory elements including destroying the original ESE and generating a new ESS, ultimately leading to abnormal splicing (Figure 2A).

### Splicing Assay *in vivo* and *vitro*

**Figure 3:**
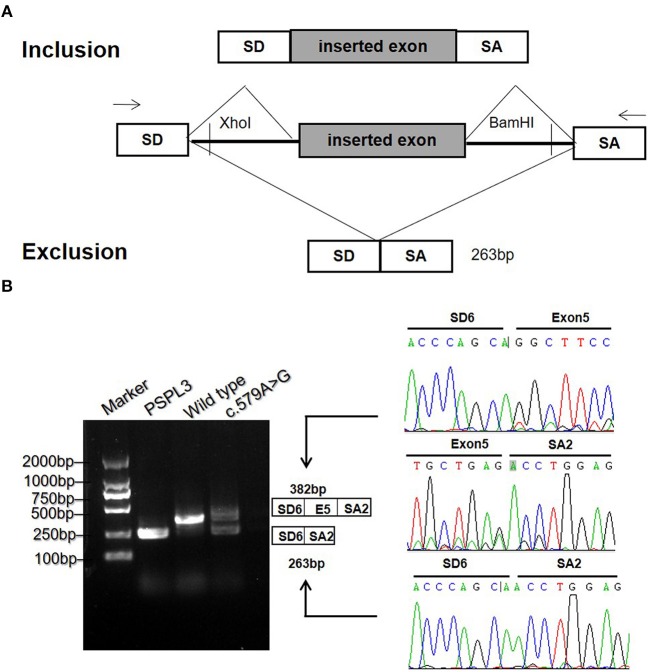
The minigene splicing assay *in vitro* for the synonymous variant c.579A>G based on the pSPL3 exon trapping vector. **(A)** The pSPL3 vector contains 2 exons SD and SA, and a functional intron. All of the indicated fragments were separately cloned into the XhoI and BamHI cloning sites of the pSPL3 vector. SD6 and SA2 primers were designed for RT-PCR amplification of cDNA sequences generated by transfected HEK293T cells. **(B)** Electrophoresis and sequencing of RT-PCR products of wild type and mutant c.579A>G allele observed two species of RNA compared to one product for the wild type allele. The lower fragment resulted from the c.579A>G allele lost the exon 5.

To validate the prediction *in silico*, experiments of RT-PCR and direct sequencing of the product *in vivo* and minigene splice assay *in vitro* were conducted. The results *in vivo* showed that the patient's leukocytes expressed two transcripts that differed by about 100 bp. Sequence analysis of the RT-PCR products showed that the shorter transcript lacked a sequence corresponding to exon 5 (Figure 2B). The minigene splice assay demonstrated that c.579A>G could cause an exon skipping event of exon 5 in the *ETFDH* mRNA transcript, probably leading to a truncated protein (Figure 3). Eventually, the mutation c.579A>G was rated as pathogenic based on the ACMG guidelines. These two mutations were submitted to the LOVD database.

## Discussion

MADD is a group of lipid storage myopathies whose clinical manifestations are highly variable and related to the onset period. In the late-onset type, MADD may manifest beyond the neonatal stage as isolated muscle weakness and myofibril destruction because of intracellular lipid deposits (3). However, many muscle disorders, such as inflammatory myopathies, metabolic myopathy, and progressive muscular dystrophy also present with muscle weakness. Thus, MADD may be misdiagnosed as a different type of lipid storage myopathy, a glycogen storage disease, Guillain-Barré syndrome, or other muscle disease (8). As a non-invasive inspection, tandem mass spectrometry has been highly applied in pediatrics for patients with no specific clinical features, such as our patient. MADD could be diagnosed by tandem mass spectrometry, even during asymptomatic phases of the disease (9). As MADD is the most common disease of fatty acid oxidation defects in China (10), we suggest that tandem mass spectrometry may be a first-tier test for muscle weakness beyond the muscle biopsy for Chinese.

MADD is caused by defects in the alpha (ETFA) and beta (ETFB) subunits of electron transfer flavoprotein (ETF), and electron transfer flavoprotein dehydrogenase (ETFDH) (1). In this report, we described a rare case of late-onset MADD with a pathogenic synonymous variant in the *ETFDH* gene. Synonymous mutations are still rarely reported in human diseases (11, 12). This underreporting is likely to be caused by the perception that synonymous mutations were silent without modifying the amino acid sequence of a protein and by the absence of functional studies to evaluate their pathogenicity. However, growing studies remind us that they can cause changes in protein expression and function, by affecting mRNA splicing (13). In this report, bioinformatics and mRNA analysis suggested that the synonymous variant c.579A>G disrupted exonic splicing regulatory sequences. These short nucleotide sequences within exons bind splicing regulatory proteins to regulate alternative splicing. Efficient splicing has limited tolerance of mutations in the exonic splicing regulatory sequences, even if they have no effect on protein coding, which explains the abnormal RNA processing observed in our study. The skipping of mutant exon 5 in the RNA transcript was expected to result in a frameshifted ETFDH protein. Thus, it is necessary to investigate synonymous variants by bioinformatics analysis and functional studies when searching for causative mutations.

The accurate diagnosis benefits the treatment of our patient. Consistent with previous reports, supplementation of riboflavin, the precursor of flavin adenine dinucleotide (FAD), has improved muscle weakness of our patient harboring *ETFDH* variants (14). The *ETFDH* gene contains 13 exons and encodes a 617 amino acid ETFDH protein, which acts in conjunction with ETF to govern electron transport from several FAD-containing acyl-CoA dehydrogenases to the main respiratory chain in mitochondria. FAD is a cofactor for ETFDH protein which is important for the enzyme catalytic activity, correct folding, and protein stability (14). Riboflavin supplements probably increase the intra-mitochondrial FAD concentration and ameliorate the effects of mutations that reduce the affinity of ETFDH protein for FAD (14). Especially, the therapeutic application of antisense oligonucleotide (ASO) is very promising for patients with mutations disrupting the mRNA processing. ASOs are short synthetic oligonucleotides that can bind to pre-RNA through Watson-Crick base pairing and modulate pre-RNA splicing in the target gene bypassing the disease-causing mutation. Currently, several ASOs have received approval by the U.S. Food and Drug Administration for the therapy of neurological disorders (15). Our patient may also benefit from ASO-based therapy in the future who carry an *ETFDH* synonymous variant causing exon skipping.

In summary, we reported a rare case of MADD resulting from two novel pathogenic mutations in the *ETFDH* gene. The synonymous variant c.579A>G, once thought to be silent, was validated *in vivo* and *vitro* to cause exon skipping. Understanding the effect of synonymous variants could have important implications in the pathogenicity and therapeutic insights of MADD.

## Data Availability Statement

The datasets generated for this study can be found in the LOVD database with Individual ID 00263297.

## Ethics Statement

The studies involving human participants were reviewed and approved by the ethics committee of the Children's Hospital of Nanjing Medical University. Written informed consent to participate in this study was provided by the participants' legal guardian/next of kin. Written informed consent was obtained from the individual(s), and minor(s)' legal guardian/next of kin, for the publication of any potentially identifiable images or data included in this article.

## Author Contributions

GH and JZ performed the clinical and laboratory data collection and wrote the manuscript. CW, WZ, and BZ performed the molecular diagnostics. BZ, JY, and ZJ contributed the important intellectual content during manuscript drafting and revision. Text revision was performed by all authors. All authors read and approved the final manuscript.

### Conflict of Interest

The authors declare that the research was conducted in the absence of any commercial or financial relationships that could be construed as a potential conflict of interest.
